# Potential Role of Flavivirus NS2B-NS3 Proteases in Viral Pathogenesis and Anti-flavivirus Drug Discovery Employing Animal Cells and Models: A Review

**DOI:** 10.3390/v14010044

**Published:** 2021-12-28

**Authors:** Abdul Wahaab, Bahar E Mustafa, Muddassar Hameed, Nigel J. Stevenson, Muhammad Naveed Anwar, Ke Liu, Jianchao Wei, Yafeng Qiu, Zhiyong Ma

**Affiliations:** 1 Shanghai Veterinary Research Institute, Chinese Academy of Agricultural Science, Shanghai 200241, China; wahaaabwahaaab@gmail.com (A.W.); muddassarh@vt.edu (M.H.); dr.naveed903@gmail.com (M.N.A.); liuke@shvri.ac.cn (K.L.); jianchaowei@shvri.ac.cn (J.W.); 2 Sub Campus Toba Tek Singh, University of Agriculture, Faisalabad 36050, Pakistan; bahar.mustafa@uaf.edu.pk; 3 Department of Biomedical Sciences and Pathobiology, College of Veterinary Medicine, Virginia Polytechnic Institute, State University, Fralin Life Sciences Building, 360 W Campus Blacksburg, Blacksburg, VA 24061, USA; 4 Royal College of Surgeons in Ireland, Medical University of Bahrain, Busaiteen, Adliya 15503, Bahrain; n.stevenson@tcd.ie; 5 Viral Immunology Group, School of Biochemistry and Immunology, Trinity Biomedical Sciences Institute, Trinity College Dublin, D02 R590 Dublin, Ireland

**Keywords:** flaviviruses, NS2B-NS3 proteases, genome organization, pathogenesis, characterization, antiviral drug target, in vitro and in vivo models

## Abstract

Flaviviruses are known to cause a variety of diseases in humans in different parts of the world. There are very limited numbers of antivirals to combat flavivirus infection, and therefore new drug targets must be explored. The flavivirus NS2B-NS3 proteases are responsible for the cleavage of the flavivirus polyprotein, which is necessary for productive viral infection and for causing clinical infections; therefore, they are a promising drug target for devising novel drugs against different flaviviruses. This review highlights the structural details of the NS2B-NS3 proteases of different flaviviruses, and also describes potential antiviral drugs that can interfere with the viral protease activity, as determined by various studies. Moreover, optimized in vitro reaction conditions for studying the NS2B-NS3 proteases of different flaviviruses may vary and have been incorporated in this review. The increasing availability of the in silico and crystallographic/structural details of flavivirus NS2B-NS3 proteases in free and drug-bound states can pave the path for the development of promising antiflavivirus drugs to be used in clinics. However, there is a paucity of information available on using animal cells and models for studying flavivirus NS2B-NS3 proteases, as well as on the testing of the antiviral drug efficacy against NS2B-NS3 proteases. Therefore, on the basis of recent studies, an effort has also been made to propose potential cellular and animal models for the study of flavivirus NS2B-NS3 proteases for the purposes of exploring flavivirus pathogenesis and for testing the efficacy of possible drugs targets, in vitro and in vivo.

## 1. Introduction

**Figure 1:**
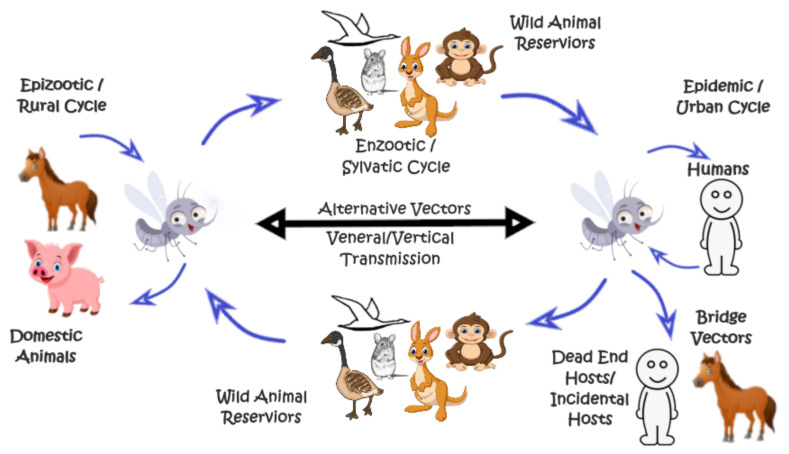
Typical life/transmission cycle of arthropod-borne Flaviviruses.

The genus, Flavivirus (family *Flaviviridae*), consists of more than approximately 70 viruses, out of which the majority are arthropod-borne viruses, including dengue virus (DENV), Japanese encephalitis virus (JEV), Zika virus (ZIKV), and West Nile virus (WNV) [1,2,3,4]. They are so named because they were found to be associated with the causation of yellow fever in humans (the Latin word “flavus” means “yellow”) [3,4]. More than twenty kinds of flaviviruses are responsible for the causation of a myriad of zoonotic diseases. Arthropod-borne flaviviruses are usually cycled among widely diverse avian and mammalian hosts (Figure 1) [5,6,7,8,9,10,11,12,13,14]. Viral persistence is a staple for pathogenesis and is maintained, predominately, without any obvious detrimental effects on the host biology. Their replication and persistence are also well documented in cell cultures [15,16,17,18,19]. Approximately 40 spp. of flaviviruses are responsible for a variety of diseases in humans. Many of these viruses are capable of causing high mortality and morbidity rates [20]. The family comprises four main genera, which include Pestivirus, Pegivirus, Hepacivirus, and Flavivirus. Within each genus, the virus may be subdivided into various antigenic groups, based on the serology or the molecular phylogeny, and categorized into different clusters, clades, and subspecies [21]. Morphologically, flaviviruses have a size of approximately 500 A° and consist of the RNA genome (positive-sense single-stranded RNA that is linear), enclosed in a capsid, which is further surrounded by an envelope. RNA is infectious in nature. The approximately 11 kb (although the genome length varies in different members) genome of flaviviruses encodes a single open reading frame (ORF) and untranslated regions (UTRs), which are present at 5′ and 3′ of the genome. The ORF contains three basic structural proteins (SPs) and seven nonstructural proteins (NS proteins). The SPs are located at the 5′ end of the RNA genome, and they include: a core protein, also known as a nucleocapsid (C protein); an envelope protein (E protein), which is often glycosylated, and that is also a major antigen that is subjected to neutralization by antibodies; and a nonglycosylated membrane protein (M protein). The nonstructural proteins (NS1, NS2A, NS2B, NS3, NS4A, NS4B, and NS5), which are present at the 3′ end of the genome, have a variety of functions, and are primarily involved in RNA replication, virus assembly, and the modulation of host responses [22,23,24]. Among the NS proteins, only NS3 and NS5 are known to perform a variety of enzymatic reactions. NS3 encodes the RNA helicase [25,26] serine protease [24,27], RNA triphosphatase (RTPase) [27,28], and nucleocapsid triphosphatase (NTPase) activities [29,30,31]. In particular, NS2B and NS3 are mainly responsible for performing the proteolytic cleavage in the virus (the remainder of the cleavage is performed by the proteases from the cellular origin [23,32,33,34]), in addition to encoding guanyl and methyltransferase (GTase and MTase activities) [35,36,37]. Flaviviruses replicate inside macrophages, monocytes, and dendritic cells, and they tend to replicate in the cytoplasm of the host cell in order to induce a variety of cytopathic alternations in the cells [38,39]. Regardless of the genus, the attachment of the virus to the cells is almost always mediated by the E protein [14,40,41]. The phenomenon of receptor-mediated endocytosis is manipulated for viral entry into the cells [41,42,43,44]. The low pH of the endosome triggers the fusion of the host cell membrane and the virus, causing the release of RNA in the cytoplasm of the cell. Once inside the cell, the cytoplasm is the site where viral replication takes place [45]. However, it is important to mention here that flaviviruses do not completely stop the host cell’s RNA and protein synthesis [46,47]. The assembly of the virion has been observed to occur in the endoplasmic reticulum (ER), as well as in the cell membrane in the case of mosquito cells. The final release of the virion occurs through exocytosis [48]. The subviral particles (SVPs) (without genome and capsid protein) consist only of a lipid bilayer, along with bound prM-E complexes, and are produced as a byproduct of the viral assembly process. After final processing and release from the ER, these SVPs are released as whole noninfectious particles [49]. The functional and structural insights of flavivirus proteases summarized in this review may advance our current knowledge of flavivirus replication and accelerate the efforts for the development of vaccines and/or broad-spectrum antivirals against flaviviruses.

## 2. Structure and Role of NS3 and NS3 Protease Domain in Flavivirus Replication

**Figure 2:**
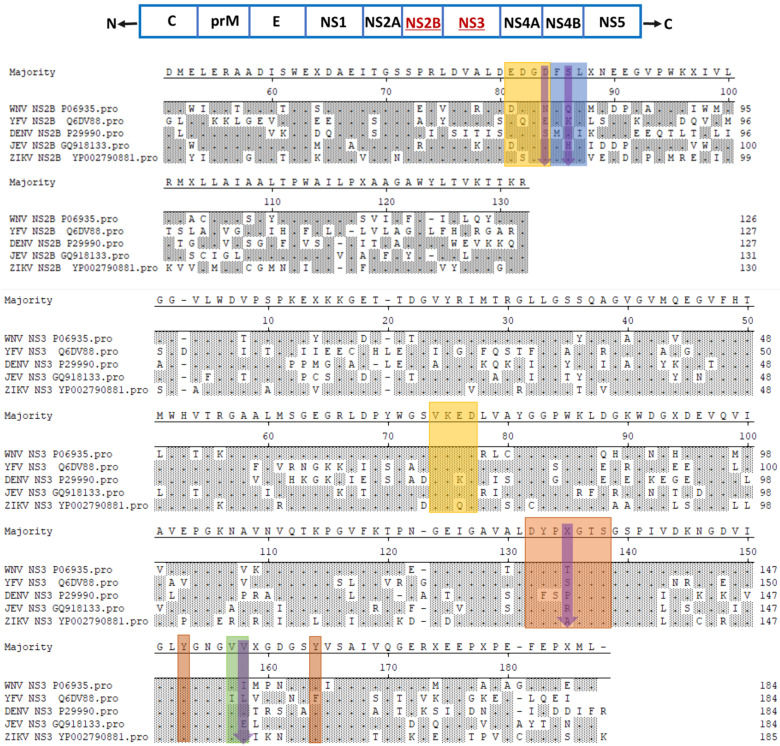
Multiple sequence alignment of NS2B/NS3 protease from different flaviviruses (WNV, YFV, DENV2, JEV, and ZIKV). Residues located in four distinct substrate-binding pockets, i.e., S1, S2, S3, and S4, marked in orange, yellow, cyan, and green, respectively [55,74]. Nonconserved residues located at the binding pockets are marked in magenta arrowheads [75].

The genetic similarity between the members of the flavivirus genus predicts the main features necessary for the viral replication cycle [50]. The NS3 is one of the major viral proteins possessing enzymatic function. It is found to be the most conserved among the viral proteins, and it exhibits approximately 65% sequence identity among JEV, WNV, DENV, YFV, and ZIKV (Figure 2) [51]. As described previously, there are two major domains of this protein, which are the RNA helicase and protease domains, connected through a short linker (flexible). The three-dimensional structure of NS3 proteins has been well documented and resolved for various flaviviruses. However, depending on the virus replication stage, different conformations can exist [51,52]. For instance, the binding of RNA is one event that can induce a conformational change [53,54]. The N-terminal domain of the flavivirus NS3 protein consists of protease domains that contain four homologous sequences to serine protease. Three of them form catalytic domains, whereas the fourth helps in substrate binding [55]. It has been suggested that the specificity of the substrate binding is because of an aspartic acid residue, which is located in the lower portion of the binding pocket [56]. The exact site for the proteolytic cleavage depends on the cleavage site sequence, and it may vary among different members; however, the majority of these sites contain two basic residues, which are followed by a side chain within the viral polyprotein [57].

## 3. Structure and Role of NS2B and NS2B Hydrophilic Domain in Flavivirus Replication

NS2B consists of approx. 130 amino acids, and is a type of small integral membrane protein, having a molecular weight of 14 kD. It consists of three hydrophobic domains (which are supposed to be part of the transmembrane domain) and a central hydrophilic domain [58,59]. Studies have suggested that the central hydrophilic domain is required for the activation of NS3, and that any mutations in it can cause the defective protease activity of NS3, or may even cause NS3 instability, leading to faulty viral assembly [58,60,61,62,63,64,65]. NS2B (H) (hydrophilic domain of NS2B) essentially acts as a cofactor for the protease activity of the NS3 protein. The initial characterization of the cofactor requirement for various flaviviruses has revealed that the minimal essential region for protease activity is positioned in a 40–50 residue central hydrophilic segment of NS2B (amino acid 45 to 95) [32,58,66,67]. NS2B contains a hydrophilic region, the central region of which contains a β-barrel, which folds around the β-barrel of the NS3 protease for its stability [55]. Upon substrate binding, conformational changes occur in the NS2B (C-terminal domain), which leads to the stability of β- hairpin, which becomes the component of the active site [55,68]. The active NS2B (H)-NS3 protease is essential for the cleavage at the NS2A/NS2B, NS2B/NS3, NS3/NS4A, and NS4B/NS5 junctions [69,70]. Moreover, it has also been proposed that the cleavage of capsid protein may also be mediated through the NS2B-NS3 protease [71]. The NS2B-NS3 interaction may also cause the tethering of NS3 at the membrane, causing replicase complex anchoring at the compartment membranes [72]. Both NS2B(H) and NS3 are associated with the membrane structures (virus-induced) [73]. This suggests that the interaction of NS3 with NS2B (H) is mandatory for its membrane localization [63].

## 4. Dengue Virus (DENV)

A deletion analysis of NS2B in DENV has demonstrated the sufficient role of the central hydrophilic region as a cofactor of NS3 [66,76,77]. The dengue virus (DENV) possesses a polyprotein that is needed to be processed, and that has been found to undergo cleavage at the rER of the host by NS2B-NS3 (cytoplasmic side) and by host cell peptidase (luminal side) [24]. NS2B (a.a. 1394 to 1440) is required as a cofactor for NS3 protease (a.a. 1476 to 1660) [77] and is also involved in the recognition of the substrate [78]. In the dengue virus, NS2B often acts as a cofactor of NS3 (protease domain), and it consists of 130 amino acids (15 kDa) [79]. The N and C terminal domains are located in the cytoplasm. It is proposed to have a helical bundle that consists of approximately four alpha-helix subunits (1–4), which are short and transmembrane. Between the α2 and α3 subunits, it contains a central hydrophilic domain (consisting of 40 residues and that is highly conserved), which is responsible for its cofactor activity [80]. This domain leads to heterodimerization with the NS3 protease domain (noncovalently), and it results in the formation of a functional membrane-bound protease complex. This complex is needed for the appropriate localization and activation of the serine protease. There are suggestions that it is also needed for the trimerization of NS2B-NS3, although the exact mechanism remains to be elucidated [81]. In the open conformation of NS2B-NS3, the catalytic site is not wrapped by the cofactor, while in the closed conformation, it is needed for the appropriate recognition of the substrate, as well as for efficient proteolysis. Moreover, the latter is also the most predominant form of NS2B-NS3 in the solutions, whether it is ligand-bound or not [81,82]. The protease activity of the NS2B-NS3 leads to viral protein cleavage at NS2A/NS2B, NS2B/NS3 (through cis-cleavage), NS3/NS4A, and NS4B/NS5 (through trans-cleavage). This protease complex is also needed for the internal cleavage within the NS2A, NS4A, and NS3 helicases. Cleavage occurs at the dibasic motifs (RR, KR, RK) at P1 and P2, and at a short chain amino acid at P1′ (A, G, or S). The protease complex also cleaves the C protein at the C terminus (at dibasic motifs, which are conserved) [63,82]. Interestingly, while studying the noncofactor roles of NS2B, it has been shown that there is colocalization of the NS2B with dsRNA, which indicates that it might be a part of the replication complex [83]. Moreover, it has also been implicated in viral replication, its assembly, and release, and thus may contribute towards the cytopathic effects (in combination with NS2A) [84]. In DENV, the oligomerization of the NS2B with the host cell membrane may be mediated by its alpha-helical TMD (transmembrane domain). In human red blood cells (RBCs), the DENV NS2B has been shown to destabilize and increase the membrane permeability that leads to pore formation [85]. NS2B mutations at the Trp 62 residue resulted in the complete elimination of the cis-cleavage ability of the NS2B-NS3 protease, while the substitution of alanine at Leu 75, Ile 77, and Ile 79 resulted in reduced proteolytic activity [86]. Recently, it has also been shown that NS2B (alone, or with NS3) interferes with type 1 interferon (IFN) production. This is conducted by specifically targeting the cyclic GMP-AMP synthetase (cGAS) for degradation. cGAS is required for binding with DNA (self or nonself) in the cytoplasm, and it activates a series of biochemical changes through signal transduction that ultimately results in STING activation, which is required for type 1 IFN generation. DENV NS2B causes the degradation of cGAS through the autophagy/lysosomal mediated pathway [87].

## 5. Yellow Fever Virus (YFV)

The yellow fever virus genome contains 10862 nucleotides, which encode a long precursor polyprotein. At the membranes of the ER, the generation of viral proteins occurs by the cleavage of the viral polyproteins. The cleavage of the viral structural proteins and NS4B (N-terminus) is mediated by signal peptidase, while the cleavage of the NS1-NS2A is mediated by the host protease (membrane-bound) in the host cell [88]. The cleavage of the remaining capsid protein (membrane-anchored), as well as the cotranslational cleavages, are mediated by the NS3 protease along with the NS2B cofactor [71,79,89,90,91]. The various cleavage sites include consensus (C/virion C, 2A/2B, 2B/3, 3/4A, 4A/2K, and 4B/5) and alternative sites (aAα) [92]. The N-terminal of the NS3 protein possesses a trypsin-like serine protease domain that preferentially cleaves the two adjacent basic amino acids, e.g., RR or KR, or, in some cases, QR, QK in the consensus sequence of G/ARR2S/G [58,61,92]. The conserved central region of NS2B, and the amino-terminal region of the NS3B, together form the NS2B-NS3 protease complex. Just as in DENV, the NS2B-NS3(pro) constitutes a stable complex that mediates the polyprotein substrate cleavage, both in the cis and the transform [58]. However, charged amino acids are important for this protein cleavage, as it has been determined that the mutations involving charged-alanine replacement at NS2B–NS3181 have demonstrated that they affect polyprotein processing [93].

## 6. Zika Virus (ZIKV)

Structural studies have shown that the NS2B-NS3 protease of ZIKV exists in two forms: a closed form and an open form. In the presence of a substrate or inhibitor, it usually adopts a closed conformation, while in the absence of the substrate or inhibitor, it is in open conformation [94,95]. It has been shown that NS2B surrounds the NS3 in such a way that it leads to the formation of β-hairpin, which then makes an important contribution to the formation of the S2 pocket of NS3 [94,95,96]. The NS2B of the Zika virus exhibits a higher level of disorderliness, especially from the 62–98 residue region (37 residues) [97,98]. Ultimately, NS2B interacts with NS3 in such a way that it leads to the cleavage of the polyprotein into a variety of functional proteins, which are important in viral replication and maturation [55].

## 7. Japanese Encephalitis Virus (JEV)

Japanese encephalitis (JE) is a vaccine-preventable disease caused by the Japanese encephalitis virus (JEV), which is primarily prevalent in Asia. The JEV is classified into a single serotype, with five genetically distinct genotypes, i.e., I, II, III, IV, and V, having an 11 Kb genome, comprising three structural and seven nonstructural proteins [99,100,101,102]. In JEV, the N-terminal 1/3rd (180 residues) of the NS3 contains protease active sites, which include His 51, Asp 75, and Ser 135 [103]. Just as in other flaviviruses, NS2B acts as the cofactor of the NS3 serine protease [58,66]. NS2B-NS3 proteases have been involved in carrying out a variety of important phases, e.g., RNA replication (viral), polypeptide cleavage, and the processing and assembly of viral particles [104,105]. The protease activity of the JEV NS2B/NS3 leads to the viral polyprotein cleavage of the capsid (internal), NS2A/NS2B, NS2B/NS3, and NS3/NS4A sites [32]. Moreover, NS2B-NS3 proteases may also play an important role in the immune evasion by the virus [105]. It has been shown that NS2B-NS3 proteases have been involved in the cleavage of interferon stimulators. In mice, this ability was found to play a critical role in enhanced viral replication, as well as in enhanced virulence [106]. Researchers have also demonstrated that certain mutations in the NS2B-NS3 region (NS2B-99, NS3-78, and NS3-177) contribute to the enhanced infectivity of JEV (genotype I) in amplifying hosts [107]. In JEV, the residues, Ser 46 to Ile 60 (in particular Trp 53, Glu 55, and Arg 56), are essential for the NS3 protease activity (both cis- and trans-activity), just as in DENV4 and YFV. The NS2B of JEV is found to exhibit 67% similarity with the WNV NS2B sequence, while it is found to exhibit 28–34% with other mosquito-borne flaviviruses [108].

## 8. West Nile Virus (WNV)

**Figure 3:**
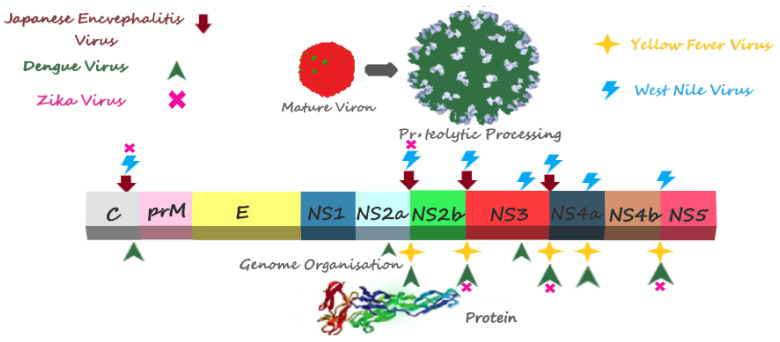
Cleavage sites proteolytically processed by NS2B-NS3 proteases in polyproteins of various flaviviruses: Cleavage sites proteolytically processed by Japanese encephalitis virus NS2B-NS3 proteases are shown by red arrows [32]; West Nile virus NS2B-NS3 cleavage sites are shown by blue lightning [67,118,122,123]; Yellow fever virus NS2B-NS3 cleavage sites are shown by yellow stars [69,70,124]; Dengue virus NS2B-NS3 cleavage sites are shown by green arrows [70,76,79,82,92,125,126]; and Zika virus NS2B-NS3 cleavage sites are shown by pink Xs [127].

In the West Nile virus, just as in other flaviviruses, NS2B (25 kDa) consists of a transmembrane protein (hydrophobic) that is involved in the replication of the genome, the formation of the membranous structure, and the assembly of virions [109,110]. In order to obtain the association of the protease complex into virus-induced membranes, the domains at both the N and C terminals (residues at 59–62 and 75–87, respectively) play an important role [111]. Another study has shown that the mutation in NS2B at D(80)DD and G83 results in a reduction in the viral NS2B-NS3 protease activity, as well as replication [112]. The unwinding activity of RNA by NS3 is likely made possible after the association of NS2B with NS3 [73,113]. The exact mechanism by which NS2B acts as a cofactor is not completely understood; however, several studies have revealed that, in the presence of NS2B, there is a substantial rearrangement in the NS3 [55,68]. Crystal structures have shown that the NS2B (residue 49–88) tends to form a belt that surrounds the NS3 protease domain. This interaction then forces the NS3 to adopt active conformation [55,77,109]. The NS3 protein is a highly conserved protein that possesses serine protease activity at the N-terminal domain. As this protein lacks a transmembrane domain, after its cleavage from polyprotein, it either goes in the cytoplasm, or remains retained in the ER, where its enzymatic domains are needed [114,115]. Just as with other flaviviruses, it is only active in the presence of the NS2B cofactor, and, in the case of its absence, the NS3 protease domain remains inactive [66,79,116]. This complex (NS2B(H)-NS3) then cleaves the viral polyprotein into a variety of structural and nonstructural proteins [111,117]. The complex of NS2B-NS3 proteases has been found to localize within the convoluted membranes (CM) or para crystalline (PC) arrays, which suggests the possible involvement of the membranes in the proteolytic cleavage [78]. In WNV, the proteolytic activity of the NS3 (Pro), in association with NS2B (hydrophilic region; residue 50–97), has been demonstrated by employing an *E. coli* expression system [118]. Recently, crystal studies involving DENV and WNV NS2B-NS3 proteases have demonstrated that the residues, 51–57 and 82–85 of the NS2B, are important for the stabilization of the NS3 protease and the substrate recognition activity, respectively [55]. Sequence analysis and mutation studies have revealed that the determinants of the flavivirus NS2B protein (except in JEV), which control NS3 protease activation and activities, are located at the positions: Glu52-Leu53-Lys54- Lys55 of YFV [62,93]; Trp62, Leu75-Ser76-Ile77-Thr78-Ile79, and Glu89-Glu90-Glu91- Glu92 of DENV-2 [86,119]; and Trp60, Gly68, Gln77, Gly81, and Val88 of Alkhurma virus (ALKV) [120]. It has also been reported that the NS2B-NS3 proteases were responsible for the apoptosis in human medulloblastoma cells through the activation of caspase-3 and the mitochondrial mediated pathway [121]. The cleavage sites, which are proteolytically processed by the NS2B-NS3 proteases in the polyproteins of various flaviviruses, are summarized in Figure 3.

## 9. Interaction of Flavivirus NS2B-NS3 Proteases with Cellular Proteins

The Flaviviral RNA tends to replicate on the membrane of the ER, leading to the formation of a replication complex. Many cellular and viral factors participate and are pivotal for the formation of this complex. Therefore, several NS proteins (including NS2B/NS3 proteases) of the flaviviruses act together to retain the replication assembly at the ER. Owing to the larger genome of the DENV, extensive interactions are needed between DENV and the host cells. It has been reported that the NS3 protein of DENV redirects the fatty acid synthase (FASN) on the ER (the replication site for DENV). It was also seen that DENV-infected cells demonstrated the increased synthesis of the fatty acids during infection [128]. Moreover, it was also found that Rab 18 (GTPase located in the ER and that is responsible for vesicle trafficking) helps in the DENV replication by recruiting FASN to the sites where the virus is replicating, and by facilitating its interaction with NS3 to trigger fatty acid synthesis [129]. Recently, it has been reported that the NS3 of DENV (full-length isolated helical and protease domains of NS3) also interacts with the glyceraldehyde-3-phosphate dehydrogenase (GAPDH) enzyme, and this results in enhanced NS3 ATPase activity and reduced glycolytic activities [130]. The nonspecific functions of GAPDH are mRNA translation and stability [131,132]. Therefore, it may be postulated that the interaction between NS3 and GAPDH may result in the unwinding of double-stranded (ds) RNA, as well as vesicle formation (vesicle-induced), which is ultimately needed for virion assembly [130]. Recently, it has been found that the JEV NS3 protein also interacts with the isoforms of the 14-3-3 protein (14-3-3 ɛ and 14-3-3 η) to block the translocation of the RIG-1 and MDA-5 from the cytosol to the mitochondria, thereby suppressing the host immune response, leading to enhanced viral replication in the cells. The researchers further postulated that the 14-3-3 protein is well conserved among insects, humans, and mice, and that targeting it may thereby facilitate viral replication in multiple hosts [133,134].

In order to maintain homeostasis, cells perform the process of reticulophagy (in which they degrade ER). FAM134B is one of the important host cell restriction factors located on the ER. A study has shown that the NS3 proteases of WNV, DENV, and ZIKV can cause the cleavage of FAM134B, thereby suppressing the reticulophagy pathway, leading to enhanced viral replication, presumably by utilizing the ER membrane for efficient viral budding [135]. The ZIKV NS2B/NS3 protease is also involved in interactions with many other cellular proteins, which include the cleavage of the cytoskeletal factor, Septin-2 (at residue R306), which results in slow cell division, enhanced apoptosis, multipolar spindles in the mitotic defects, and delayed cytokinesis in the neural progenitor cells (NPCs). These changes are likely to produce microencephalopathy [136]. Another study recently indicated that the DENV NS2B-NS3 protease is involved in the cleavage of the DDX21 protein. DDX21 is an RNA helicase enzyme that is involved in the host cell defense against a myriad of viral infections. In cells infected with DENV, there is a translocation of DDX21 from the nucleus to the cytosol. This causes the activation of IFN-β, and thus inhibits the DENV replication during early viral replication. Thus, DENV NS2B-NS3 proteases cause the subversion of the host cell immune system in order to facilitate enhanced viral replication [137]. While the interactions of NS3 with many cellular target proteins result in enhanced viral replication, many other proteins interact with the virus in a way that results in reduced viral replication. A heat shock protein, (Hsp 40) DNAJB6, interacted with NS3, which resulted in reduced viral propagation [138].

Mitochondria and mitochondrial-associated membranes (MAMs) are also known to play an important role in several processes that are pivotal for viral replication, i.e., ATP generation, lipid synthesis, and the induction of cellular apoptosis [139]. Flaviviruses also interact with mitochondria and MAMs and can regulate (up or down) these processes, causing the disturbance in cellular homeostasis. A recent study has demonstrated that the DENV NS2B3 protease interacts with mitochondria and results in the cleavage of MAMs and microfusion (MFN1 and 2) that ultimately leads to the fragmentation of the mitochondria, which can contribute to disease pathogenesis [140]. Keeping this in view, another study was designed to investigate the NS3 protease location in mitochondria. It was found that the N-terminal of the NS3 protease bears a mitochondrial signal sequence, and this facilitates its localization in the matrix of the mitochondria. Upon viral entry into the mitochondria, it was found that the NS3 pro and NS3 pro helicases both resulted in the cleavage of the GrpEL1 protein; the finding was also observed in the samples of clinically infected patients. GrpEL1 protein functions as a cochaperon of the Hsp-70 protein, which implies that the cleavage of the GrpEL1 protein may lead to the dysfunction of the Hsp-70 protein. The exact consequences of this dysfunction are yet to be elucidated; however, based on the correlation between the cellular level of the GrpEL1 protein and the platelet count, the possible dysfunction of the mitochondria was postulated, which leads to thrombocytopenia [141].

## 10. Interactions of Flavivirus NS3 with Host Cell NPC and Nucleus

The majority of macromolecular transport between the nucleus and the cytoplasm is mediated mainly through the nuclear pore complex (NPC). The NPC is a disk-like structure (500 nm × 100 nm) that consists of multiple copies of 30 different proteins, which are termed “nucleoporins” (Nups). The NPC and its associated machinery play a pivotal role in the regulation of many cellular pathways. Different viruses have evolved a variety of strategies in order to manipulate the NPC in such a way that ultimately leads to the favoring of viral replication in cells [142]. Altering the NPC integrity is also one of the major activities carried out by viral-encoded proteases to facilitate the viral entry into the nucleus, thus favoring viral replication. This phenomenon has not only been observed in viruses replicating in the nucleus, but also in viruses that replicate in the cytoplasm. The flaviviral proteins are known to interact with the NPC and the associated proteins to disrupt the nucleocytoplasmic trafficking, and to gain entry into the nucleus [139]. The latter strategy may be adopted so that the NPC changes result in the reduced trafficking of mRNA or other transcription factors, which can result in a suppressed immune response against that viral infection [143]. Recent studies have also demonstrated the ability of flaviviral NS2B-NS3 to affect the integrity and distribution of nucleoporins (Nups). Nup62, Nup98, and Nup153 have been found to be disrupted by DENV, whereas Nup98 and Nup153 were affected by the ZIKV NS2B-NS3 proteases [144]. These studies indicate that the NPC and the associated factors in host cells are manipulated as the targets for Flaviviridae replication.

The NS3 of ZIKV tends to locate itself in the perinuclear regions of the infected cells, and causes alterations in the nuclear lamina structure, which leads to the formation of extrusion sites. This may affect the function of centromeres [145]. It has also been observed that NS3 tends to deposit itself on the concave surface of the nucleus (kidney-shaped altered nuclei) and may also be involved in changing the other components of the nuclear envelope [146]. Other studies have indicated that the NS3 of DENV is located on the nucleus of infected cells at an earlier time (8–12 h) than on cytoplasm (16–24 h), postinfection [147,148].

## 11. Characterization of Flavivirus NS2B-NS3 Proteases

In order to design an appropriate flavivirus inhibitor, the very first approach is to design an appropriate substrate and optimize the in vitro reaction/working conditions for viral NS2B/NS3 proteases. Various substrate profiling studies have shown that the WNV protease preferentially cleaves at the K/R motifs. The presence of bulky residues, e.g., Tyr, Trp, or Phe at positions P1 or P2, can be well tolerated by the DENV protease as long as the Gly occupies the other position [108,149]. The sequences of amino acid required for polyprotein processing in DENV, WNV, and YFV are homologues; however, minor differences exist among them. In DENV, the hydrolysis sites exist after a pair of basic residues, e.g., Lys-Arg, Arg-Arg, or Arg-Lys at positions P1 and P2 [69]. In WNV, the majority of cleavage sites possess Lys and Arg sequences at positions P2 and P1, and Gly at P1′ [150]. Importantly, the YFV polyprotein processing sites contain a pair of Arg-Arg, followed by Gly, Val, or Ser [89]. The substrate sites/sequences susceptible to cleaving by various flavivirus NS2B-NS3 proteases are summarized in Table 1.

The pH, the buffers, and the reaction temperature are crucial to characterizing flaviviral NS2B/NS3 proteases [76,77]. Several researchers have optimized these conditions to efficiently determine the in vitro proteolytic activities of NS2B-NS3 proteases, which are compiled in Table 2.

## 12. NS2B-NS3 Proteases as a Potential Viral Inhibition Drug Target

Flavivirus two-component nonstructural NS2B-NS3 proteases are essential for the viral life cycle and, consequently, are a promising drug target. Using NS2B-NS3 proteases is one of the major antiviral strategies for researchers. Just as in HIV and HCV, protease offers a unique target for the inhibition of viral replication by employing a variety of peptides and pseudopeptides [167]. Substrates with di- or polybasic recognition sequences exhibit a strong affinity for viral protease. This recognition tends to be conserved among various Flaviviruses and, therefore, it may be employed as a promising antiviral target with a relatively broad spectrum [61]. The shallowness of the substrate-binding pocket, and its exposure to the solvents, make the interaction of the protease and the peptidomimetics labile. Moreover, the stability and permeability of the peptidomimetics are further hindered by the polybasic residues at P1 and P2. The other possible strategy may disrupt the interaction between the NS2B and NS3 domains [168]. Numerous studies have employed the in silico (molecular docking) approach or have used high-throughput chemical screening for the discovery of novel NS2B-NS3 protease inhibitors [169,170]. Moreover, substrates having fluorogenic peptides have also been used for the discovery of novel NS2B-NS3 proteases inhibitors. The active protease was produced in a bacterial expression system, and the enzyme’s specificity for synthesized FRET-type substrate libraries was profiled [171]. These protease inhibitors may be categorized as peptides and are also known as “peptidomimetics” (substrate-derived) or “small molecules” (not substrate-derived). Peptides/peptidomimetics exhibit high affinities and minimal drug-like molecules, whereas the latter ones act in a reverse manner, i.e., they have less affinity and are more drug-like. The desirable lower nanomolar range of the dissociation constants (in association with protease) is only exhibited by very few inhibitors, and a majority of them are peptide-based substrate mimetics [172,173,174,175,176]. Numerous antivirals have been screened against flaviviruses targeting recombinant viral proteases, the details of which are provided in Table 3.

## 13. Proposing Role of STING in Development of In Vitro and In Vivo Models for Studying Flavivirus Pathogenesis and Antiviral Drug Screens

One of the major strategies used to develop a vaccine or antiviral drug against flavivirus is through studying disease by employing animal models (Table 4). However, it is difficult to use such models to study flaviviral pathogenesis and disease control measures. For instance, various studies have reported using humanized mice for studying the clinical infection of DENV, with several limitations associated with their use [204,205,206,207,208,209,210,211,212]. There is also a paucity of information on using them to successfully test DENV and ZIKV vaccines [213]. The lack of animal models against flaviviruses has hampered a deep understanding and the development of novel therapeutics/vaccines against most of the flaviviruses. To be used for vaccine or novel therapeutic testing, animal models must exhibit immune competency and viremia (reproducible) against the virus. Moreover, it is also required that the animal model inoculated with a particular virus must demonstrate the same signs as in natural infection. For example, in the case of DENV, none of the humanized mice exhibited the classical features of hemorrhage and the leakage of plasma [214]. That is why a combination of several different models is needed to test the therapeutic efficacy of a novel antiviral or vaccine candidate against different flaviviruses.

Finding a cellular protein that acts particularly as a substrate for some enzymes greatly increases the mechanistic specificity for that protein. Studies in the past have demonstrated a potential new cellular target, the STING (the stimulator of interferon gene) protein, which may allow researchers to develop some appropriate animal models to design novel therapeutics against flavivirus NS2B-NS3, as it has been found that all flavivirus NS2B-NS3 (except YFV) preferentially cleaves to the STING as a substrate [215].

The STING is a multipass protein that resides on the ER, and it plays a pivotal role in inducing the innate immune signaling upon intracellular infection [216,217,218,219]. Originally, it was proposed that it is activated on the intracellular binding of cytosolic DNA species, such as viral DNA, [217,220]. However, later studies have demonstrated that it may also be activated by viral RNA infection [221]. Because of its important role in innate immunity and interferon (IFN) production, several viruses possess proteins that can degrade the STING [215,222,223,224,225]. The NS2B-NS3 proteases of flaviviruses (WNV, ZIKV, JEV, and DENV; but not YFV), for instance, effectively cleave the STING in human cells, leading to the lower production of type I IFN by those cells, resulting in enhanced intracellular viral replication [215,222,225,226]. Moreover, DENV is also known to play a critical role in the degradation of STAT2, another player in the host immune response [227,228,229,230]. However, the mice STING is resistant to degradation by flavivirus proteins, which results in strong interferon responses and protects them from flavivirus infection [222,225,227,231]. For this reason, they are unable to be used as an effective model for experimental flavivirus infection.

In order to study the flavivirus NS2B-NS3 proteases in vitro, it is very important to develop cellular models that have functional STINGs that may be cleaved by flavivirus NS2B-NS3 proteases, as it occurs under clinical circumstances in humans. A variety of cells from the human lineage may be used for this purpose. A recently conducted study on ZIKV demonstrated the ability of ZIKV-associated NS2B-NS3 proteases to cleave the STING in fibroblasts derived from humans, as well as nonhuman primates (NHPs) [215]. The results from this study make it possible to use NHP-derived fibroblasts as a possible cell-based model to study and develop novel antiviral drugs/vaccines against flavivirus NS2B-NS3 proteases in vitro.

Similarly, in most of the NHPs, DENV cannot degrade the STING because of a small variation in the STING sequence of nonhuman primates [226]. This small variation may demonstrate the reason for better DENV replication in humans. Further studies have shown that the STING can effectively be degraded in three species of rodents and apes, each indicating the possibility of using these species as an effective model of flavivirus replication in the nonhuman host [226]. The results of this study are promising and provide new hope for the use of these animal hosts as models for studying pathogenesis, and for designing novel therapeutic products against flavivirus NS2B-NS3 proteases.

Moreover, testing drugs on animals prior to humans is one of the preliminary requirements. The abovementioned studies may also pave the path towards finding a possible use for NHPs as animal models for studying the pathogenesis of different flaviviruses. Therefore, in the future, different NHPs (Old and New World monkeys, great apes) may be tested for different flavivirus replications, thus allowing for the use of in vivo models for flavivirus replication, understanding pathogenesis, and devising novel antiviral treatments.

## 14. Conclusions

In this review, the structure, optimized reaction/working conditions, potential antiviral targets, and possible cellular and animal models are proposed to study the NS2B-NS3 proteases of various flaviviruses. One approach to treat flavivirus infection is through developing enzyme inhibitors. This approach involves finding compounds that can interact and disorient the enzymatic active site in such a way that it is no longer capable of carrying out its specific function/reaction. Thus, this approach often serves as the starting point for selecting an antiviral inhibitor, whose binding affinity for the active site often resembles, or even exceeds, the normal substrate. Using this approach, many promising compounds have been discovered, as described previously. However, it is also important to mention here that, despite more than two decades of research, not much success has been observed in developing NS2B-NS3 protease inhibitors, and there are several reasons. Firstly, the flat and hydrophobic nature of the enzyme’s active site greatly hinders the strong binding affinity of the inhibitor with its active site. Secondly, from a toxicological point of view, the structure of the active site of NS2B-NS3 proteases greatly resembles the host serine proteases, and, thus, the use of such NS2B-NS3 protease inhibitors may lead to severe damage in the host cells. Therefore, prolonged studies must be conducted at the cellular level and with experimental animals before its consideration for use in humans. Moreover, as the active site exhibits great affinity towards positively charged substrates/inhibitors, the use of such compounds may have some negative effects on the bioavailability of the compounds.

The latest crystallographic studies of NS2B-NS3 proteases with substrate-bound and unbound forms have provided some mechanistic evidence of the enzymatic mode of action, which may help in the future for developing a potential safe inhibitor of NS2B-NS3 proteases. Both in silico and high-throughput screening (HTS) methods may be deployed initially for shortlisting the inhibitors of NS2B-NS3 proteases, which may later be confirmed through crystallographic studies. These studies may help in identifying the more allosteric sites in flavivirus NS2B-NS3 proteases, and may lead to the discovery of more effective, potent, and safe flavivirus NS2B-NS3 protease inhibitors. Moreover, as the structures of NS2B-NS3 proteases exhibit great similarity in different flaviviruses, efforts must be made to find an antiviral agent that can be used effectively to inhibit proteases from different flaviviruses, and that thus exhibit a broad range of antiviral activities.

## Figures and Tables

**Table 1:** Cleavage sites from various flaviviruses: Arrowheads indicate the NS2B-NS3 protease-susceptible cleavage positions in the polyproteins of various flaviviruses.

Flavivirus	Cleavage/Substrate Sites					Reference
	Capsid C	NS2A/NS2B	NS2B/NS3	NS3/NS4A	NS4B/NS5	
JEV	VNKRGRKQNKRJ **↓**GGNEGS	NPNKKR **↓**GWPATE	LKTTKR **↓**GGVFWD	FAAGKR **↓**SAISFI	KPSLKR **↓**GRPGGR	[151]
	NKRGRKQNKR **↓**GGNEGSIMWL	GLMVCNPNKKR **↓**GWPAT EFLSA	GYWLTLKTTKR **↓**GGVFWDTPSP	WFKDFAAGKR **↓**SAVSFIEVLG	-	[32]
YFV	LSSRKRR **↓**SHDVLT	RIFGRR **↓**SIPVNE	VRGARR **↓**SGDVLM	FAEGRR **↓**GAAEVL	MKTGRR **↓**GSANGK	[152]
WNV	INBBSTKQKKS **↓**GGTAGF	OPNRKR **↓**GWPATE	LQYTKR **↓**GGVLWD	FASGKR **↓**SQIGLV	KPGLKR **↓**GGAKGR	[153,154,155,156]
	-	DPNRKR **↓**GW	LQYTKR **↓**GG	FASGKR **↓**SQ	KPGLKR **↓**GG	[122]
ZIKV	KERKRR **↓**GADTSIGI	TRSGKR **↓**SWPPSEVL	VKTGKR **↓**SGALWDVP	FAAGKR **↓**GAALGVME	GLVKRR **↓**GGGTGETL	[127,157]
DENV1	MNRRKR **↓**SVTMLL	-	-	-	-	[158]
DENV2	LNRRRR **↓**TAGMII	RTSKKR **↓**SWPLNE	EVKKQR **↓**AGVLWD	FAAGRK **↓**SLTLNL	-	[159]
DENV3	INKRKK **↓**TSLCLM	-	-	-	-	[160]
DENV4	LNGRKR **↓**STITLL	KGASRR **↓**SWPLNE	QVKTQR **↓**SGALWD	FASGRK **↓**SITLDI	AQTPRR **↓**GTGTTG	[161,162]

**Table 2:** In vitro reaction conditions for the optimum proteolytic activities of various flavivirus NS2B-NS3 proteases.

Flavivirus	Optimum Buffers and Reaction Conditions					Reference
	Tris-HCl	NaCl	Glycerol	Temp	pH	
DENV	50 mM	50 mM	35%	37 °C	8.5	[67]
JEV	50 mM	25 mM	30%	37 °C	9.5	[75]
WNV	200 mM	13.5 mM	30%	37 °C	9.5	[163]
ZIKV	20 or 50 mM	150 mM	10 or 20%	37 °C	8.5	[50,164,165]
	**Tris-HCl**	**Acetic Acid**	**Glycine**	**Temp**	**pH**	
YFV	75 mM	25 mM	25 mM	37 °C	7.0	[166]

**Table 3:** Antivirals and their mechanisms screened by targeting flavivirus two-component NS2B-NS3 proteases.

Sr No	Flavivirus	Antivirals Screened by Targeting NS2B/NS3 Proteases	Mechanism	Reference
1	WNV (West Nile Virus)	Benzoyl-norleucine-lysine-arginine-arginine (Bz-nKRR) tetrapeptide aldehyde	C-terminal electrophile incorporation	[177]
		Cationic tripeptides (along with nonpeptide cap)		[176]
		Peptide–boronic acid inhibitors		[173]
		Benzyl ethers of 4-hydroxyphenylglycine	N-terminal capping moiety optimization	[172]
		Bz-Arg-Lys-X-NH		[178]
		Peptide-hybrids based on 2,4-thiazolidinedione scaffolds containing nonpolar groups		[179]
		Benzyl ethers of 4-hydroxyphenylglycine	P1 and P2 basic residue modulation	[172]
		Aprotinin	Noncompetitive inhibitors	[117]
		Palmatine (Coptis chinensis)		[180]
		Derivatives of Guanidinylated 2,5-dideoxystreptamine	Competitive inhibitors	[181]
		Benzoyl-norleucine-lysine-arginine- arginine (Bz-nKRR) tetrapeptide aldehyde	Aldehydic inhibitors	[177]
		Cationic tripeptides (along with nonpeptide cap)		[176]
		Aprotinin	Stearic hindrance of active site	[175]
		D-arginine-based 9–12-mer peptides	Mechanism yet to be determined	[175]
		Furin		[182]
		C-Terminal Electrophile incorporation	Peptide–boronic acid inhibitors	[173]
2	DENV (Dengue Virus)	Tetrapeptide: Bz-Nle-Lys-Arg-Arg-B(OH)2 (boronic acid analogue)	C-Terminal electrophile incorporation N-terminal capping moiety optimization	[170]
		Benzyl ethers of 4-hydroxyphenylglycine		[172]
		Bz-Arg-Lys-X-NH	N-terminal capping moiety optimization P1 and P2 basic residue modulation	[178]
		Rhodanines and Thiazolidinediones		[183]
		Benzyl ethers of 4-hydroxyphenylglycine		[172]
		Plectasin	Noncompetitive inhibition	[184]
		Substitution of Arg with unnatural Arg motifs in the P2	P1 and P2 basic residue modulation Aldehydic inhibitors(against DENV 2)	[185]
		Benzoyl-norleucine-lysine-arginine- arginine (Bz-nKRR) tetrapeptide aldehyde		[177]
		Cationic tripeptides (along with nonpeptide cap)	Aldehydic inhibitors (against DENV 2)	[176]
		Cyclopentapeptide (CKRKC)	Mechanism yet to be determined	[186]
		BP-2109		[187]
		BP13944		[188]
		BT 24 (quinoline compound)		[189]
		Aminobenzamide		[190]
		2,5,6-trisubstituted pyrazine compounds		[191]
		Furin		[182]
		Protegrin-1		[192]
		Retrocyclin-1		[193]
		Chalcone derivatives (DENV-2)		[194]
		Flavonoids (fingerroot) (DENV-2)		[194]
		Tyrothricin	Competitive inhibition	[195]
		Derivatives of Guanidinylated 2,5-dideoxystreptamine		[181]
		Retrotripeptides: R-Arg-Lys-Nle-NH2 Ivermectin Selamectin Benezethonium chloride	Mixed inhibition	[196] [195]
		Peptide-boronic acid	C-terminal electrophile incorporation	[173]
3	ZIKV (Zika Virus)	Peptidomimetic boronic acid	Formation of salt bridge with Asp83 of NS2B	[95]
		Bromocriptine	Mechanism yet to be determined	[197]
		Novobiocin		[198]
		Hydroxychloroquine		[199]
		Erythrosin B		[200]
		Theaflavin-3,3′-digallate		[201]
		9b (HIV protease inhibitor)		[202]
		2,5,6-trisubstituted pyrazine compounds		[191]
		Aprotinin		[75]
4	JEV (Japanese Encephalitis Virus)	NSC135618	Inhibits the conformational change of NS2B (allosteric inhibitor)	[203]
5	YFV (Yellow fever Virus)	Erythrosin B	Mechanism yet to be determined	[200]

**Table 4:** Animals and cellular models for studying Flavivirus pathogenesis/vaccine development.

Animal Models for Studying Dengue Virus (DENV)					
Animal Type	Model	Study Conducted/Findings			Reference
Nonhuman Primates	Rhesus macaquesa	Inactivated vaccine (DENV-II).			[232]
		Expression of G protein in Vaccinia virus (DENV-2).			[233]
		DNA vaccine (encoding Pr-M and E) of DENV-2.			[234]
		DENV-I vaccine.			[235]
		Tetravalent vaccine expressed in Adenovirus.			[236]
		Tetravalent DNA vaccine (chimeric).			[237]
		Mutant DENV (live attenuated) vaccine.			[238]
		Inactivated DENV (tetravalent).			[239]
		DNA vaccine.			[240]
	Cynomolgous macaques	Live attenuated and recombinant vaccine comparison.			[241]
		Chimeric DENV1/2 vaccine.			[242]
		Recombinant DENV.			[243]
		Recombinant protein (DENV 1–4).			[244]
		Tetravalent DENV vaccine (chimeric).			[245]
		Tetravalent DENV vaccine (live attenuated).			[246]
		DENV-2 virus-like particles.			[247]
Mice	A/J	DENV-2 caused thrombocytopenia.			[248]
	AG129 (do not have type I and II Interferon receptors)	DENV caused neurological manifestations leading to death.			[249]
		DENV infection caused systemic infection and vascular leakage, leading to death.			[250]
		DENV infection resulted in splenomegaly.			[251]
	IFNAR−/− (Lack of IFN type I receptors; background of C57BL/6 mice)	DENV-2 infection resulted in viral growth in small intestine, liver, and bone marrow, resulting in death.			[252]
	Cardif −/−	DENV infection in mice resulted in viral growth in lymph nodes, bone marrow, and spleen.			[253]
	STAT 1 −/−	DENV infection resulted in viral growth in kidney, liver, and small intestine; however, the mice survived.			[254]
	STAT 2 −/−	DENV infection resulted in viral growth in kidney, liver, and small intestine; however, the mice survived.			
	STAT 1 −/− STAT 2 −/− (Lack STAT 1 and 2 proteins)	DENV infection resulted in higher viral titers in serum, kidney, liver, small intestine, and spleen, and mice death occurred.			
	STAT1−/−/ IFNAR−/− (Lack of STAT1 and type I IFN receptor)	DENV infection resulted in higher viral titers in serum, kidney, liver, small intestine, and spleen, and mice death occurred.			
	STAT1−/−/ IFNGR−/− (Lack of STAT1 and type II IFN receptor)	Mice survived			
**Animal Models for Studying Yellow Fever Virus (YFV)**					
**Animal Type**	**Model**	**Study Conducted/Findings**			**Reference**
Nonhuman Primates	Cynomolgous macaques	YFV-DENV(1–4) vaccine			[255]
		YFV-DENV Chimeric vaccine			[256]
**Models for Studying Flavivirus NS2B-NS3 Proteases**					
**Virus Type**		**Cells**	**Animal Spp.**	**Outcome**	**Reference**
DENV ZIKV JEV WNV		Dermal fibroblasts (DFs)	Great apes (Pan paniscus, Pan troglodytes, Pongo pygmaeus Gorilla gorilla)	Dermal fibroblasts (DFs) demonstrated increased mice susceptibility to infection by Flaviviruses.	[215]
			Old World monkeys (Macaca nemestrina, Papio anubis, Macaca mulatta)	Increased mice susceptibility to infection by Flaviviruses.	
			New world monkeys (Saimiri sciureus)	Increased mice susceptibility to infection by Flaviviruses.	
			Mice (Tmem173Gt)	STING disruption increased mice susceptibility to infection by Flaviviruses; however, they could not develop serious infection (underlines the role of redundant pathways in viral replication dynamics).	

## Data Availability

Not applicable.
